# The marker of alkyl DNA base damage, N7-methylguanine, is associated with semen quality in men

**DOI:** 10.1038/s41598-021-81674-x

**Published:** 2021-02-04

**Authors:** B. Altakroni, C. Nevin, M. Carroll, C. Murgatroyd, G. Horne, D. R. Brison, A. C. Povey

**Affiliations:** 1 Centre for Occupational and Environmental Health, Epidemiology and Public Health Group, Division of Population Health, Health Services Research and Primary Care, School of Health Sciences, Faculty of Biology, Medicine and Health, Manchester Academic Health Sciences Centre, University of Manchester, Manchester, M13 9PL UK; 2 Department of Life Sciences, Faculty of Science and Engineering, Manchester Metropolitan University, John Dalton Building, Chester Street, Manchester, M1 5GD UK; 3 Department of Reproductive Medicine, Old Saint Mary’s Hospital, Manchester Academic Health Science Centre, Manchester University NHS Foundation Trust, Oxford Road, Manchester, M13 9PT UK; 4 Division of Developmental Biology & Medicine, School of Medicine, Maternal and Fetal Health Research Centre, Faculty of Biology, Medicine and Health, University of Manchester, Manchester, M13 9PL UK

**Keywords:** Biomarkers, Stem cells

## Abstract

Sperm DNA contains a range of DNA base damage that can arise, in part, from exposure to methylating agents. However, the effects are not fully characterized and so the aim of this study was to investigate associations between semen quality and the levels of N7-methyldeoxyguanosine (N7-MedG), a marker of exposure to methylating agents, and other markers of DNA damage and DNA methylation. Sperm samples were collected from 105 men attending an assisted reproduction clinic as part of a couple undergoing treatment for infertility and semen quality assessed manually according to WHO guidelines. Semen levels of N7-MedG, quantified by immunoslotblot, were significantly higher in men with sperm concentration < 15 × 10^6^/ml (p ≤ 0.01), semen volume < 1.5 ml (p ≤ 0.05) and also in men with any aspect of semen quality below WHO reference levels (p ≤ 0.001). Measures of neutral Comet DNA damage were correlated with semen quality in a univariate analysis but not after adjustment for N7-MedG levels. Sperm concentration was negatively associated with % methylation at the gene for *DAZL* but no other marker of global or gene-specific DNA methylation. Results support the hypothesis that the known toxic and DNA damaging properties of alkylating agent exposure may have direct deleterious consequences on semen quality.

## Introduction

Sperm DNA contains damage that may impact on semen quality and ART outcome^1,2^. This damage is not fully characterised, with the most widely used assays measuring some loss in DNA integrity such as single and/or double strand DNA breaks, DNA fragmentation or DNA denaturation^3^. In addition, specific DNA base damage has also been detected in sperm DNA, particularly that arising from reactive oxygen species such as 8-oxoguanine^4^. However, other types of DNA base damage including that arising from methylating agents has also been reported^5^. The potential adverse consequences of exposure to DNA methylating and other alkylating agents on somatic cells i.e. toxicity, mutagenicity and carcinogenicity is well recognised^6^. Such changes are largely ascribed to DNA damage formation^7^, though exposure to alkylating agents can induce other cellular changes including changes in gene expression^8^ or histone phosphorylation^9^. Treatment of male mice with the ethylating agent, *N*-ethyl-*N*-nitrosourea, causes mutations in sperm that has led to the development of many models of human disease^10,11^. In contrast very little is known about DNA alkylation in human spermatozoa . We reported several years ago that N7-MedG levels, a biomarker of exposure to methylating agents^12^, were associated with male factor infertility in a population of men attending as a part of a couple for ART^5^. N7-MedG is quantitatively the most significant methyl DNA adduct, constituting around 70–85% of all adducts produced by methylating agents^13^. Though N7-MedG does not alter DNA duplex structure or coding properties and is neither mutagenic nor toxic^14^, N7-methylguanine in DNA is relatively unstable and can depurinate leading to abasic sites that are toxic and mutagenic^15^ and can further lead to DNA strand breaks^16^. More recently it has been reported that N7-MedG adducts can form reversible, but toxic, DNA–protein cross links with histone proteins^17^. Furthermore, overexpression of the DNA repair protein, N3-methylpurine-DNA glycosylase, that repairs this adduct, sensitises cells to the genotoxic effects of methylating agents^14,18^. In addition, the presence of N7-MedG in DNA, depending upon the type of alkylating agent exposure, may also indicate significant levels of the pro-mutagenic, pro-carcinogenic and teratogenic adduct, *O*^6^-methylguanine^19^.

Associations between DNA alkylation and other types of DNA damage or DNA modifications, such as epigenetic methylation, in human sperm are unknown. To provide further information on these associations with semen quality, we have investigated these markers in sperm of men attending an infertility clinic as part of a couple.

## Results

The study included 105 semen samples with data available for semen quality, DNA damage measured by Comet assay, and N7-MedG (101). Of these, 44 also had DNA methylation data.

### Descriptive analysis of semen quality and markers of DNA damage and DNA methylation

The semen quality of the studied population is shown in Table 1 together with a descriptive analysis of markers of DNA damage and DNA methylation. There were 28 men whose sperm concentration was less than 15 × 10^6^/ml, 18 men with a % progressively motility of < 32, and 11 men whose semen volume was less than 1.5 ml. Overall there were 44 men with a below reference level of either sperm concentration, % progressive motility or semen volume.

**Table 1:** Descriptive analysis of semen quality and markers of DNA damage and DNA methylation.

Variable	Value	Mean ± SD (n)	Range
Age	Years	35.3 ± 5.8 (105)	26–57
Semen quality	Sperm concentration (× 10^6^/ml)	59.1 ± 66.2 (105)	0.5–362.0
	Progressive motile sperm (%)	49.0 ± 17.1 (105)	1.0–77.0
	Immobile sperm (%)	40.9 ± 14.5 (105)	0.5–99.0
	Semen volume (ml)	3.2 ± 1.4 (105)	0.5–8.1
Comet assay	Median tail DNA (%)	1.02 ± 0.76 (105)	0.07–5.28
	Sperm with low DNA damage (LDD) (%)	15.4 ± 8.3 (105)	2.3–39.2
	Sperm with high DNA damage (HDD) (%)	14.7 ± 8.6 (105)	4.0–47.2
N7-MedG	fmoles/μg DNA	0.55 ± 0.35 (101)	0.12–2.30
Global methylation	nM	396.1 ± 71.3 (44)	296–687
Gene Methylation	DAZL (% methylated)	4.6 ± 5.0 (31)	0.4–20.0
	H19 (% methylated)	40.4 ± 11.9 (44)	12.8–73.0
	MEG3 (% methylated)	77.4 ± 27.8 (45)	0–91.3
	SNRPN (% methylated)	3.2 ± 2.5 (43)	0.7–12.3

% of sperm with low DNA damage (% LDD sperm) was inversely correlated (r_s_ = − 0.31, p < 0.001 n = 105) with % of sperm with high DNA damage (% HDD sperm) and with median % tail DNA (r_s_ = -0.79, p < 0.001 n = 105) whereas % HDD sperm was positively correlated with median % tail DNA (r_s_ = 0.67, p < 0.001 n = 105). N7-MedG in sperm DNA was negatively associated with % LDD sperm (r_s_ = -0.22, p < 0.05 n = 101) and positively associated with median % tail DNA (r_s_ = 0.20, p < 0.05 n = 101): it was not correlated with % HDD sperm (r_s_ = 0.12, n = 101). % *DAZL* methylation was positively associated with % HDD sperm (r_s_ = 0.37; p < 0.05) and with median % tail DNA (r_s_ = 0.39; p < 0.05). There was no other association between global methylation and gene-specific methylation or markers of DNA damage.

### Relationship between semen quality and markers of DNA damage and DNA methylation

Sperm concentration was negatively associated with N7-MedG levels (r_s_ = -0.32, p < 0.001), % HDD sperm (r_s_ = -0.41, p < 0.001), median % tail DNA (r_s_ = -0.31, p < 0.001) and with % methylation of *DAZL* (r_s_ = -0.56, p < 0.001) but not with global methylation or % methylation of *H19CTCF6*, *SNRPN*, or *MEG3* (Table 2). The multiple regression model including N7-MedG, %HDD sperm, median % tail DNA and % *DAZL* was not significant (R^2^ = 0.20, p = 0.2) with *DAZL* methylation showing a non-significant trend (p = 0.08: Table 3).

**Table 2:** Correlations between markers of DNA damage and methylation and semen quality.

DNA marker	Specific variable	*r*_*s*_ (n)^a^			
		Sperm concentration (× 10^6^/ml)	Progressively motile sperm (%)	Immotile sperm (%)	Semen volume (ml)
Alkylation	N7-MedG (fmoles/μg DNA)	-0.32*** (101)	-0.19^‡^ (101)	0.11 (101)	-0.12 (101)
Integrity	% sperm with low DNA damage (LDD)	0.11 (105)	0.34*** (105)	-0.31** (105)	0.09 (105)
Integrity	% sperm with high DNA damage (HDD)	-0.41*** (105)	-0.50*** (105)	0.41*** (105)	0.16 (105)
Integrity	% tail DNA (median)	-0.31*** (105)	-0.48*** (105)	0.41*** (105)	0.04 (105)
Methylation	Global methylation (nM)	0.03 (44)	0.07 (44)	-0.02 (44)	0.06 (44)
	DAZL (% methylated)	-0.56*** (31)	-0.04 (31)	0.06 (31)	0.27 (31)
	H19 (% methylated)	-0.02 (44)	-0.03 (44)	0.04 (44)	-0.06 (44)
	MEG3 (% methylated)	0.01 (45)	0.10 (45)	-0.07 (45)	0.08 (45)
	SNRPN (% methylated)	-0.02 (43)	0.05 (43)	-0.06 (43)	-0.06 (43)

^a^ *r*_*s*_ Spearman rho correlation coefficient; *p < 0.05; ** p < 0.01; ***p ≤ 0.001; ^‡^ p = 0.053.

**Table 3:** Multiple regression analysis of semen quality with DNA damage and DNA methylation markers.

Semen parameter	Model variable	B	SE B	β
Sperm concentration	Constant	132.5	42.4	
	DAZL	-4.27	2.32	-0.33^‡^
	N7MeG	-45.86	74.91	-0.12
	HDD	-0.33	2.34	-0.04
	Median tail DNA	-12.44	26.91	-0.14
Progressive motility	Constant	62.80	6.84	
	N7MeG	-7.83	3.86	-0.18*
	HDD	-0.79	0.30	-0.38**
	Median tail DNA	-1.56	4.96	-0.06
	LDD	0.26	0.26	-0.14
Immotile sperm	Constant	45.6	5.21	
	HDD	0.93	0.26	0.55***
	Median tail DNA	-8.00	3.74	-0.42*
	LDD	-0.66	0.23	-0.38**

^‡^ p < 0.1; * *p* < 0.05; **p < 0.01; ***p < 0.001.

% progressively motile sperm was positively associated with % LDD sperm (r_s_ = 0.34, p < 0.001), but negatively associated with % HDD sperm (r_s_ = -0.50, p < 0.001) and median % tail DNA (r_s_ = -0.48, p < 0.001). There was no other correlation between semen quality and global methylation or gene-specific DNA methylation (Table 2). The multiple regression model with % LDD sperm, % HDD sperm, median % tail DNA and N7-MedG produced R^2^ = 0.28, (p < 0.001) with %HDD sperm (p < 0.01) and N7-MedG (p < 0.05) being significant (Table 3).

% immotile sperm was negatively associated with % LDD sperm (r_s_ = -0.31, p < 0.01), but positively associated with % HDD sperm (r_s_ = 0.41, p < 0.001) and median % tail DNA (r_s_ = 0.41, p < 0.001). There was no other correlation between semen quality and global methylation or % methylation of *H19CTCF6*, *SNRPN*, or *MEG3* (Table 2). The multiple regression model with %LDD sperm, % HDD sperm and median % tail DNA produced R^2^ = 0.19, (p < 0.001) with %HDD sperm (p < 0.01), %LDD sperm (p < 0.01) and median % tail DNA (p < 0.05) all being significant (Table 3). Semen volume was not associated with any of the measured biomarkers.

### Associations between semen quality and DNA damage and DNA methylation in men with semen quality above and below WHO referent values

To assess whether DNA damage and DNA methylation levels were associated with clinically more relevant factors, we examined whether levels were different in men whose semen quality was either below or above WHO reference values (Table 4). Specifically, N7-MedG levels, % HDD sperm and median % Tail DNA were higher in those men whose sperm concentration was < 15 × 10^6^ ml. In contrast, % LDD sperm was lower and % HDD sperm and median %tail DNA higher, in men with < 32% progressively motile sperm. Furthermore, N7-MedG levels were higher in those men with a semen volume was less than 1.5 ml. Overall, in any men whose sperm concentration or % progressively motile sperm or semen volume was below the reference category, N7-MedG levels, % HDD sperm and median %tail DNA were significantly higher and % LDD sperm significantly lower compared to men whose semen quality was above the reference value. In this analysis there was no association between any DNA methylation marker and semen quality above or below the WHO reference value.

**Table 4:** Associations between markers of DNA damage and semen quality categorised by WHO reference values.

Semen variable	Category	Marker of DNA damage or methylation. Mean ± SD (n)			
		N7-MedG	% LDD sperm^a^	% HDD sperm^b^	% Tail DNA
Concentration	> 15 × 10^6^/ml	0.49 ± 0.29 (77)	16.2 ± 8.7 (77)	13.4 ± 8.2 (77)	0.88 ± 0.62 (77)
	< 15 × 10^6^/ml	0.75 ± 0.47 (24)**	13.3 ± 7.1 (28)	18.1 ± 8.9 (28)**	1.39 ± 0.97 (28)***
Progressive motility	> 32%	0.53 ± 0.33 (86)	16.3 ± 8.5 (87)	13.0 ± 6.9 (87)	0.87 ± 0.54 (87)
	< 32%	0.70 ± 0.46 (15)‡	11.0 ± 6.1 (18)*	22.7 ± 11.3 (18)***	1.74 ± 1.16 (18)***
Semen volume	> 1.5 ml	0.51 ± 0.28 (91)	15.6 ± 8.5 (94)	14.6 ± 8.7 (94)	1.02 ± 0.79 (94)
	< 1.5 ml	0.91 ± 0.69 (10)*	13.6 ± 6.8 (11)	15.8 ± 7.9 (11)	1.03 ± 0.44 (11)
Any category	> Reference	0.45 ± 0.20 (61)	17.3 ± 8.9 (61)	12.2 ± 7.2 (61)	0.77 ± 0.53 (61)
	< Reference	0.71 ± 0.47 (40)***	12.9 ± 6.8 (44)*	18.1 ± 9.2 (44)***	1.36 ± 0.89 (44)***

^a^ % sperm with low DNA damage^; b^ % sperm with high DNA damage;

Statistically significant by Mann Whitney U test: ^‡^ *p* ≤ 0.1; * *p* ≤ 0.05;** *p* ≤ 0.01; *** *p* ≤ 0.001.

In a logistic regression analysis (Table 5), N7-MedG levels were significantly associated with low sperm concentration (OR 6.46, 95%CI 1.51–27.7) even after adjustment (OR 6.42, 95%CI 1.47–28.0) for other markers of DNA damage, % HDD sperm and median % tail DNA. Whilst % HDD sperm and % median tail DNA were significantly associated with having low progressive motility in a univariate analysis (Table 5), after adjustment for % LDD sperm and N7-MeG there were no significant associations. N7-MedG levels were associated with an increased risk of having low progressive motility but this increase was not significant (OR_adj_ 3.42, 95%CI 0.89–13.2). N7-MedG levels were, however, significantly associated with having a semen sample with any parameter below the WHO reference value (OR 20.7, 95%CI 2.75–155.6) and after adjustment with other markers of DNA damage (OR 19.0, 95%CI 2.41–150). No markers of DNA damage were associated with poor semen quality after adjustment.

**Table 5:** Unadjusted and adjusted^a^ associations between DNA damage marker and poor semen quality categorised by WHO reference values.

DNA damage marker	OR (95%CI)						
	Semen volume	Concentration		Progressive motility		Any category	
	Unadjusted	Unadjusted	Adjusted	Unadjusted	Adjusted	Unadjusted	Adjusted
N7MedG	7.2 (1.7–30.0)	6.46 (1.51–27.7)	6.42 (1.47–28.0)	2.80 (0.79–9.91)	3.42 (0.89–13.2)	20.7 (2.75–155.6)	19.0 (2.41–150)
% LDD sperm^b^	-	-	-	0.91 (0.84–0.98)	0.98 (0.87–1.10)	0.93 (0.88–0.98)	1.03 (0.94–1.13)
% HDD sperm^c^	-	1.06 (1.01–1.12)	0.99 (0.90–1.08)	1.12 (1.05–1.20)	1.09 (0.97–1.22)	1.10 (1.04–1.17)	1.01 (0.92–1.12)
% Tail DNA	-	2.48 (1.26–4.86)	2.33 (0.75–7.31)	4.54 (1.89–10.9)	1.54 (0.23–10.2)	4.90 (2.03–11.8)	5.17 (0.8–33.2)

^a^Adjusted for other markers of DNA damage that were significantly associated with semen quality in a univariate analysis; ^b^ % sperm with low DNA damage;^c^ % sperm with high DNA damage.

## Discussion

In this study of sperm DNA damage and DNA methylation of men attending for ART as part of a couple, the main finding was that N7-MedG levels were significantly associated with adverse semen quality even after adjustment for other markers of DNA damage as measured by the Comet assay. Measures of DNA damage assessed by the Comet assay were correlated with % progressive motility and % immotile sperm and differed in men whose semen quality was below WHO reference levels in a univariate analysis but not after adjustment for N7-MedG levels. Sperm concentration was negatively associated with % methylation of *DAZL* but no other marker of DNA methylation was associated with adverse semen quality in this study.

N7-MedG is a marker of exposure to exogenous and endogenous DNA methylating agents and has been detected in a wide range of human tissues^13^. The precise contribution of exogenous (e.g. tobacco specific nitrosamines) and endogenous (e.g. s-adenosylmethionine and other agents formed in situ through *N*-nitrosation of amines) methylating agents to N7-MedG levels is unknown. Smoking is an exclusion criterion for treatment in our NHS centre and none of the men in the study were self-reported current smokers making it very unlikely that smoking was associated with N7-MedG levels.

There is also evidence that obesity is associated with DNA damage levels in that higher levels of DNA fragmentation (ie cells that did not have a detectable nucleus but only a Comet tail as detected by the alkaline Comet assay) were reported in obese men as compare to controls^20,21^. However, we found no correlation between BMI and DNA damage levels (data not shown). However, the finding in this study that N7-MedG levels were significantly associated with adverse semen quality is consistent with our previous report that N7-MedG levels were higher in men diagnosed with male factor infertility^5^ and implicates DNA base damage, and not just DNA strand breaks, as a contributing cause of male infertility. The mechanistic basis of this association is unclear but it might be related to the known chemical instability of the N7-MedG adduct leading to DNA strand breaks^16^ and it is worthy of note that N7-MedG levels were negatively associated with %LDD sperm and positively associated with median % tail DNA. In this context N7-MedG instability might represent a previously uncharacterised exposure route to increased DNA strand breaks in human sperm. Alternatively, the association between N7-MedG and adverse semen quality may be due to toxic DNA protein cross-links^17^ or due to the presence of other more toxic adducts such as *O*^6^-methylguanine or N3-methyladenine that will be formed at the same time as N7-MedG^22^. The latter 2 adducts have as far as we can ascertain not yet been reported in human sperm DNA but their presence in sperm DNA may have more deleterious consequences as *O*^6^-methylguanine is a known pro-mutagenic adduct^19^ and N3-methyladenine can induce chromosome aberrations^23^. The post-fertilisation effects of these adducts would depend not only on its levels in individual sperm but also the repair capacity of the oocyte.

The Comet assay measures a variety of different types of DNA damage (e.g. single and double strand breaks and apurinic sites) whether induced for example, by oxidative stress, chemical modifications of DNA or DNA repair processes^24,25^. The precise contribution of these different mechanisms to DNA damage in any one sample is unknown. The neutral Comet assay has been reported to measure principally double-strand breaks as Alu1 induced DNA fragmentation was higher with the neutral Comet assay than the alkaline comet assay^26^. This, though, is controversial with other authors reporting that the neutral comet assay measures single strand nicks when the DNA remains attached to the nuclear matrix^27^. As the extent of strand breaks increases, very large stretches of DNA can become detached from the matrix and move out of the nucleus and into the gel^28^. We refer to these events as DNA damage, recognising that the neutral Comet assay measures a mix of single and double strand breaks. The relationship between DNA damage detected by the neutral Comet assay and semen quality is currently unclear. The DNA fragmentation index in sperm, as assessed by the neutral Comet assay, has been negatively associated with sperm concentration and morphology in one study^1^, and with sperm viability, motility and morphology but not sperm concentration^29^. Similarly, a weak correlation was observed between tail % intensity and % motility, % normal morphology but not sperm concentration^30^. Conversely, no associations between semen quality and % tail DNA were detected in 80 healthy male volunteers^31^. It is unclear whether these differences result from differences in analysis protocols, descriptors of DNA damage or populations studied, though in one study associations between indices of semen quality varied with how the DNA damage was characterised^32^. One additional interpretation is that the differences in associations may result from differences in other types of DNA damage e.g. N7-MedG present within human sperm.

Previous studies have reported associations between environmental exposures and/or DNA damage and sperm DNA methylation in specific genes, suggesting, at least, that DNA damage and DNA methylation share a common exposure or cause and potentially that the effects of such exposure are mediated, in part, by their epigenetic effects. Associations between reactive oxygen species and *H19-IgF2* methylation have been described^33^ and also methylation levels of *IGF2* and *KCNQ1* but not *H19*, *MEG3*, *PEG3*, *SNRPN* were associated with DNA fragmentation index^34^. We also examined but found no associations between markers of DNA damage and global DNA methylation and methylation of genes that have been previously associated with male infertility^35^. This suggests that the effects of alkylating agent exposure may not be mediated through changes in DNA methylation, but rather via the direct effects of DNA damage. The exact source/origin of DNA damage detected by the Comet assay still remains unknown but there is little evidence in the present study of these sources also impacting on the methylation of sperm. If such associations do exist, they will likely depend upon the initial exposures causing the DNA damage. *DAZL* methylation was negatively associated with sperm concentration, consistent with previous studies^36^. Higher *DAZL* methylation was found in asthenozoospermia and oligozoospermia men^37^. In contrast, no associations were detected with global methylation or methylation of other genes (*H19, MEG3, NR3C1, SNRPN*) that have previously been associated with semen quality^35^; this may be due to population or sample size differences.

Like previous studies examining associations between DNA damage and semen quality, the main weakness of this study is that it is cross-sectional in nature and so unable to determine causative associations between the measured biomarkers and semen quality. In addition, there is the possibility that the levels of N7-MedG reported here may reflect levels occurring in contaminating leucocytes and round cells (for example). However, as these samples were used for clinical purposes, numbers of other cells were routinely assessed and samples identified as containing high levels of leucocytes and round cells were omitted from the study. Furthermore, seminal round cells have been reported to be mostly immature post-meiotic germ cells^38^ and leucocytes account for maximum 5% of total number of round cells in the seminal fluid^39^. The alternative possibility that low levels of leucocytes and round cells influenced the results is considered unlikely as their N7-MedG levels would have had to have been much higher than observed here , at levels only previously observed in patients receiving methylating agent chemotherapy^13^. We are thus confident that the results herein are representative of sperm N7-MedG levels.

However, our results are consistent with current knowledge regarding the toxicological consequences of alkylating agent exposure in other tissues. This study and other studies indicate that sperm DNA contains a range of different types of DNA damage that might be associated with semen quality and have more deleterious consequences post-fertilisation.

## Materials and methods

### Study population and ethical approval

105 men of couples attending the Department of Reproductive Medicine, St Mary’s Hospital, Manchester University NHS Foundation Trust, Manchester, for assisted reproduction treatment (ART), participated in this study. All couples had provided written informed consent for research and this study had Local Ethics Committee Approval (Central Manchester REC ERP/91/078, HFEA research licence R0026) and was performed in accordance with relevant guidelines/regulations.

### Semen analysis

Participants provided by masturbation a semen sample on the day of ART treatment. After liquefaction, semen volume, sperm concentration and motility were determined manually according to WHO guidelines^40^ in the hospital by biomedical and clinical scientific staff, and according to the UK NEQAS Reproductive Sciences scheme^41^. Samples were to be used for clinical treatment and those with any significant round cell/white blood cell count or samples with leukospermia were omitted from the study. After collecting the remaining sample from the hospital, the neat semen was centrifuged to separate the sperm from the seminal plasma and an aliquot of the neat sperm pellet was directly used to determine the level of DNA damage by using the neutral Comet assay.

### Extraction of DNA from sperm

Briefly, genomic DNA was extracted from up to 4 × 10^7^ sperm cells using the Qiagen Blood and cell culture DNA Midi Kit except that the proteinase K and ribonuclease A digestion step was carried out at 4 °C overnight. The following day, 0.5 ml of 5 mM DTT solution was added to each tube, mixed and incubated again in the cold room on a rotor mixer for 1 h, followed by incubation for another 1 h at 37˚C in water bath. Following the manufacturer’s instructions, the samples were added into gravity flow columns. Eluted from the columns, DNA was isopropanol and ethanol precipitated and rapidly air dried to remove residual alcohol. The concentration of extracted sperm DNA in 1X TE buffer was measured first by Nano-drop and then by a picogreen assay^5^.

### N7-MedG slotblot analysis of sperm DNA

Immunoslotblot analysis of N7-MedG in sperm DNA has been described previously^5,12^. Methylated DNA standards were prepared by reacting CT-DNA with *N*-nitroso-*N*-methylurea. Briefly, DNA samples were sonicated (Sonicator, Bandeline electronic sonopuls, UW 2070, Probe: SH70G) for 10 s at 50% power to obtain DNA fragments of size between 400 and 1500 bp and these were incubated in alkaline solution to induce imidazole ring opening. After neutralisation, DNA samples were heat denatured and then 1 µg of DNA immobilised on a nitrocellulose membrane (Whatman: Portran, BA79, pore size 0.1 µm). Membranes were incubated first with a rabbit polyclonal antibody raised against 2,6-diamino-4-hydroxy-5-N-methylformamidopyrimidine (me-Fapy) conjugated to keyhole limpet hemocyanin which specifically detected N7-MedG in mice treated with *N-*Nitroso*-N-*methylurea^42^ and then a polyclonal goat anti-rabbit-horseradish peroxidase conjugate. Membranes were incubated with ECL reagent (Amersham Hyperfilm) and exposed to an X-ray film (Fuji) which was subsequently developed and scanned using a HP Scanjet 485. The intensity of the bands for the samples and the standards was quantified by using ImageJ program and a standard curve was generated and subsequently the levels of N7-MedG in the samples were calculated. To confirm DNA binding the membrane was stained with a propidium iodide solution and scanned with a Typhoon scanner (GE Healthcare) at 610 emission and 560 absorption wavelengths and the intensity of DNA bands in the obtained image quantified using ImageJ.

### Single-cell gel electrophoresis (Comet) assay

DNA damage was assessed by a neutral comet assay according to previous studies but with minor modifications^1,43^. Sperm (~ 2 × 10^4^) were suspended in LMP agarose and applied to a microscope slide pre-coated with 1% NMP agarose. After gel setting, the slides were immersed firstly in freshly made precooled lysis buffer (2.5 M NaCl, 100 mM EDTA, 10 mM Tris-base, 10% DMSO and 1% Triton X-100, pH 10) at 4 °C, then in fresh lysis buffer containing 10 mM DTT and finally fresh lysis buffer containing proteinase K (100 μg/ml) to lyse the cells. The slides were then equilibrated for 20 min in cold electrophoresis buffer (300 mM sodium acetate 100 mM Tris, pH9) then electrophoresis was carried out at 13 V, 120–125 mA for 1 h. After neutralisation in cold neutralization buffer (0.4 M Tris-base pH 7.5), slides were stained with SYBR gold (Invitrogen, UK) used according to the manufacturer’s instructions and were then examined using an Olympus fluorescence microscope (OLYMPUS, U-RFL-T; Filter: FITC; Camera: Q IMAGING, RETIGA-SRV, FAST 1394; Software: QCapture Pro 6.0) and around 35 images containing in total around 400 sperm (Comet) were obtained. Then, % tail DNA was determined by using TriTeK CometScoreTM Freeware version 1.5 (TriTeK Corp, US) in 400 sperm.

Prior to the analysis of the participant samples, we validated our neutral comet assay by incubating sperm with up to 30 µM H_2_O_2_ and this clearly showed that the assay was able to detect increases in DNA damage induced by a DNA damaging agent. We found a dose-dependent decrease in the % of sperm with few strand breaks (r^2^ = 0.077: < 2% at the highest H_2_O_2_ concentration ) and a dose-dependent increase in median levels of % tail DNA (r^2^ = 0.91) and % sperm with high levels of DNA damage (r^2^ = 0.90: 20% at the highest H_2_O_2_ concentration). A pilot reproducibility study also showed little variance from day to day.

### Global DNA methylation

Genomic DNA was heat-denatured and digested to nucleosides by nuclease P1 (Sigma, UK) and then alkaline phosphatase (Sigma, UK). 5-methylcytosine levels were then quantified using the Global DNA Methylation ELISA Kit (Cambridge Biosciences, UK), following the manufacturer’s instructions.

### Gene specific methylation

DNA samples underwent bisulphite conversion using the EpiTect Fast DNA Bisulphite Kit (Qiagen, UK), according to the manufacturer’s instructions. Pyrosequencing target regions were amplified using the PyroMark PCR Kit (Qiagen, UK) as follows: PCR reactions were performed in a total volume of 25 μl consisting of 12.5 μl 2 × PCR master mix, 2.5 μl 10 × CoralLoad, 0.75 μl (3 μM) of each forward and reverse primer, 2 μl (~ 10 ng) bisulfite-converted DNA and 6.5 μλ H_2_O. Primer sequences covering the target CpG regions were either purchased as pre-designed CpG assays from Qiagen UK (Supplementary data Table 1), or custom oligonucleotides were designed using the PyroMark Assay Design software (Qiagen, UK) and purchased through Life Technologies (UK) (Supplementary Table 2). Gradient PCR was initially carried out to determine the optimal annealing temperature for amplification ranging from 52 to 60 °C and cycling conditions were carried out as follows: 95 °C for 15 min; followed by 45 cycles of denaturation, 94 °C for 30 s; annealing, 56 °C for 30 s; extension, 72 °C for 30 s. Finally, an extension of 72 °C for 10 min was included. PCR products were frozen at -20 °C until pyrosequencing. PCR products were immobilised to Streptavidin Sepharose High Performance beads (GE Healthcare Life Sciences, Uppsala, Sweden), denatured and the pyrosequencing primer (Supplementary Table) was annealed to the template. Pyrosequencing was performed on a PyroMark 24 Pyrosequencing system (Qiagen). Results were analysed on the PyroMark Q24 software to determine the % methylation for each CpG site.

### Statistical analyses

Semen quality was assessed both as a continuous variable and after dividing the population into two based upon WHO reference values for semen quality^44^ (i.e. sperm concentration > 15 × 10^6^/ml, % progressive motility > 32% and a semen volume > 1.5 ml). We established the Comet thresholds blind to the semen quality status. The distribution of % tail DNA in the sperm samples was not normal with the log % tail DNA showed an almost trimodal distribution in 75% of the samples, ~ 14% of the samples showed biomodal distributions while the remaining 11% samples showed multimodal distribution. It was possible to distinguish two subgroups of sperm; sperm with low levels of DNA damage (% tail DNA < 0.005%, LDD sperm) and sperm with high levels of DNA damage (% tail DNA ≥ 7.5%, HDD sperm). Correlation (Spearmann rank analysis) and multiple regression analyses (including those markers whose associations with semen quality had a p ≤ 0.1) were conducted to examine the relationship between semen quality and markers of DNA damage and DNA methylation (mean methylation of all CpG sites analysed). This regression analysis is of limited clinical value and so biomarker levels were then compared in men whose semen quality were above or below WHO referent levels in both a univariate analysis and by logistic regression after adjustment for other DNA damage markers with P < 0.1.

## Supplementary Information

Supplementary Information.

## Data Availability

The datasets generated during and analysed during the current study are available from the corresponding author on reasonable request.
