# Supplementary material for: The marker of alkyl DNA base damage, N7-methylguanine, is associated with semen quality in men
Source: Sci Rep. 2021 Feb 4;11:3121. doi: 10.1038/s41598-021-81674-x (PMC7862252; doi:10.1038/s41598-021-81674-x)
Supplement: Supplementary file 1 — Supplementary Information. [file 41598_2021_81674_MOESM1_ESM.docx]

**Supplementary Data**

**The marker of alkyl DNA base damage, N7-methylguanine, is associated with semen quality in men**

Altakroni, B^1ⱡ^, Nevin C^2^, Carroll M^2^, Murgatroyd C^2^, Horne G^3^, Brison DR^3,4^, Povey AC^1,*^

**^1^** Centre for Occupational and Environmental Health, Centre for Epidemiology, Division of Population Health, Health Services Research and Primary Care, School of Health Sciences, Faculty of Biology, Medicine and Health, University of Manchester, Manchester Academic Health Sciences Centre, Manchester M13 9PL, UK; **^2^** Department of Life Sciences, Faculty of Science and Engineering, Manchester, Metropolitan University, John Dalton Building, Chester Street, Manchester, M1 5GD, UK; **^3^** Department of Reproductive Medicine, Old Saint Mary's Hospital, Manchester University NHS Foundation Trust, Manchester Academic Health Science Centre, Oxford Road, Manchester M13 9PT, UK; **^4^** Maternal and Fetal Health Research Centre, Division of Developmental Biology & Medicine, School of Medicine, Faculty of Biology, Medicine and Health, University of Manchester M13 9PL, UK

**^ⱡ^** Deceased

**Contents**

Table 1 Pre-designed CpG assays for pyrosequencing.

Table 2 Pyrosequencing custom oligonucleotides

**Table 1 Pre-designed CpG assays for pyrosequencing.**

| **Gene** | **Assay name** | **Amplicon length (bp)** | **Sequence to analyse** | **Number**  **of CpG sites** |
| --- | --- | --- | --- | --- |
|  | **Catalogue number** |  |  |  |
|  | **Chromosome location** |  |  |  |
| *H19* exon1 | Hs_hsa_mir_675_01_PM | 163 | GCATGGGGCGAGACCAGACTAGGCGAGGCGGGCGGGGCGGA | 5 |
|  | PM00046823 |  |  |  |
|  | Chr11:2017874-2018037 |  |  |  |
| *SNRPN* | Hs_SNURF/SNRPN_01_PM | 249 | GCACGCCTGCGCGGCCGCAGAGGCAGGCTGGCGCGC | 6 |
|  | PM00168252 |  |  |  |
|  | Chr15:25200006-25200255 |  |  |  |

**Table 2 Pyrosequencing custom oligonucleotides**

| **Gene** | **Chromosome Location** | **Forward primer** | **Number**  **CpG sites** |
| --- | --- | --- | --- |
|  |  | **Reverse primer (biotinylated)** |  |
|  |  | **Sequencing primer** |  |
| *DAZL* | Chr3:16647040-16647131 | GTGTGTTTGTGGGTTTATGTGA | 16 |
|  |  | CACCACTTCTAAAACTACTATAAAATC |  |
|  |  | TGTGGGTTTATGTGAG |  |
| *MEG3* | Chr14:101250091-101250246 | TAGAGTAGGTTTGAGAGAGTGT | 12 |
|  |  | ACATCATACAACCTAAACTTTCTACAA |  |
|  |  | TTGAGGAGGGGAGTT |  |
